# Association between HER2 status in gastric cancer and clinicopathological features: a retrospective study using whole-tissue sections

**DOI:** 10.1186/s12876-015-0384-1

**Published:** 2015-11-04

**Authors:** Renato Santos Laboissiere, Marcelo Araújo Buzelin, Débora Balabram, Marina De Brot, Cristiana Buzelin Nunes, Rafael Malagoli Rocha, Mônica Maria Demas Álvares Cabral, Helenice Gobbi

**Affiliations:** Departamento de Medicina DEMED-UFSJ, Campus Dom Bosco. Praça Dom Helvécio, 74. Fábricas, São João del-Rei, MG 36301-160 Brazil; Departamento de Anatomia Patológica FM-UFMG, Avenida Prof. Alfredo Balena, 190 - 3o. andar, Belo Horizonte, MG 30130-100 Brazil

**Keywords:** HER2, Gastric cancer, Immunohistochemistry, Gene amplification, Clinicopathological features

## Abstract

**Background:**

Gastric cancer is usually diagnosed in an advanced stage of disease and treatment options are sparse. Trastuzumab was recently approved for metastatic or locally advanced carcinomas arising in the stomach or in the gastroesophageal junction in patients with HER2-positive tumors. However, data on the frequency of HER2-positive cases among Brazilian patients are limited. Our aim was to characterize HER2 protein and gene status in a series of Brazilian patients with gastric cancer and to evaluate its association with clinicopathological data.

**Methods:**

Histological slides from 124 primary gastrectomies were reviewed and their pathological reports were retrieved from the files at a Brazilian university hospital. Automated immunohistochemistry for HER2 was performed on whole-tissue sections from each tumor. HER2-equivocal cases by immunohistochemistry were submitted to automated dual *in situ* hybridization for gene amplification evaluation. HER2 status was confronted with clinicopathological parameters in order to assess statistically significant associations.

**Results:**

Immunohistochemistry analysis revealed that 13/124 cases (10.5 %) were HER2 positive (3+), 10/124 cases (8.1 %) were equivocal (2+) and 101/124 cases (81.4 %) were negative, being 7 cases 1+. None of the equivocal cases showed gene amplification. The overall HER2 positivity rate was 10.5 %. There was an association between HER2 expression and Laurén’s intestinal histological subtype (*P* = 0.048), well to moderately differentiated tumors (*P* = 0.004) and presence of lymphovascular invasion (*P* = 0.031). No association was found between HER2 status and tumor topography.

**Conclusions:**

Confronted with data published by other authors, the lower percentage of HER2-positive cases found in our series might be partially explained by the lower frequency of tumors arising at the gastroesophageal junction in comparison with distal gastric carcinomas in Brazilian patients. This could also account for the lack of statistically significant association between HER2 status and tumor topography in our study.

## Background

About one million new cases of gastric cancer (GC) are estimated to occur each year in the world, corresponding to 8 % of all cancer diagnoses globally [1, 2]. GC is also considered the second leading cause of cancer-related deaths worldwide and over 700,000 deaths are expected to occur due to this disease annually [1, 3]. However, the distribution of patients with GC in different parts of the globe is not homogeneous: over 70 % of new cases and deaths occur in developing countries. The highest incidence rates are found in Eastern Asia, Eastern Europe, and South America [1]. The National Institute of Cancer of Brazil estimates that GC comprises over 5 % of all malignancies in this country [4].

Treatment options for GC are generally limited, since it is usually diagnosed at an advanced stage of disease and, therefore, the five-year survival rate is consistently low, around 20 % in most parts of the world [2, 5]. According to the ToGA study, a recent phase III international randomized trial, treatment in combination of chemotherapy and trastuzumab (TZB) has significantly improved survival in patients with HER2-positive advanced GC [6]. TZB is a recombinant monoclonal antibody targeting HER2, a transmembrane protein which belongs to the family of human epidermal growth factor receptors [7]. HER2 intracellular domain shows tyrosine kinase activity, capable of generating intracytoplasmic second messengers that coordinate nuclear expression of genes associated with angiogenesis, cell proliferation, and survival [8]. Overexpression of the HER2 protein, a product of a proto-oncogene situated at chromosome 17, has been associated with carcinogenesis and tumor progression of breast, ovary, salivary glands, prostate and gastrointestinal tract cancers [9].

Based on ToGA trial results, international regulatory agencies have recently approved the use of TZB in HER2-positive metastatic or locally advanced carcinomas arising in the stomach or in the gastroesophageal junction (GEJ) [10]. Carcinomas from eligible patients for treatment with TZB must present HER2-positive status, attested by protein overexpression (score 3+) by immunohistochemistry (IHC) or gene amplification by *in situ* hybridization (ISH) for tumors with equivocal IHC results (score 2+) [11, 12]. Since then, many studies have focused on presenting the frequency of HER2-positive GC in different populations, with a wide variation on methods used for protein and gene detection. Therefore, results remarkably range from 2 to 45 % of HER2 overexpression in GC series [13–17]. A large systematic review including data from 49 studies found a median rate of 18 % of HER2 positivity in 11,337 patients with GC [18]. Nevertheless, the prevalence of HER2-positive cases among Brazilian patients remains unknown, with few data published [19–26]. Since the Brazilian public health system has not yet endorsed the use of TZB for GC treatment in this country, we believe that the detection of HER2-positive GC in Brazilian patients may provide scientific basis for the establishment of new public health policies and define better clinical protocols to improve treatment of these patients in Brazil.

Therefore, in the present study, we aimed to characterize HER2 expression in GC in a series of Brazilian patients, using whole-tissue tumor sections from surgical specimens, and to evaluate the association between HER2 protein and gene status with clinicopathological data.

## Methods

The present study was approved by the Ethics Committee of our institution (Comitê de Ética e Pesquisa COEP-UFMG) under CAAE protocol number 32898114.9.0000.5149. Written informed consent was obtained in accordance with the institutional guidelines.

We studied 142 consecutive patients who underwent primary gastrectomy between 2007 and 2011 at the Clinics Hospital, Federal University of Minas Gerais, Brazil, whose histopathological diagnosis was GC. None of the patients had received neoadjuvant chemotherapy or other type of treatment for their tumor prior to surgery. All 142 cases had their pathological reports retrieved and, in order to confirm the diagnosis of GC, the original hematoxylin-eosin stained slides were simultaneously reviewed by two pathologists (RSL and MMDAC), one of which is an expert in gastrointestinal pathology (MMDAC). Clinical data (patients’ age and gender) and pathological parameters (tumor topography, maximum tumor diameter, stage, histological grade and subtype, presence of lymphovascular and blood-vessel invasion) were recorded. Tumors were divided into two groups according to gastric topography: distal and proximal tumors, the latter category included carcinomas from the GEJ. The TNM system criteria and recomendations from the 7th Edition of the American Joint Committee on Cancer Staging Manual were used for tumor staging [27, 28]. The index of glandular formation and cytologic pleomorphism were considered for histological grading into well, moderately or poorly differentiated tumors. Laurén’s histologic classification system was used for tumor subtyping [29].

New 4 μm-thickness whole-tissue sections were obtained from formalin fixed paraffin embedded tumor samples and submitted to automated IHC, using a BenchMark XT™ platform (Ventana Medical Systems, Arizona, USA) and the prediluted primary rabbit monoclonal antibody anti-HER2/neu (4B5 clone). All slides were subsequentially counterstained with hematoxylin. A pre-tested HER2-positive breast cancer sample was used as external positive control. Eighteen cases did not contain enough tumor tissue for subsequent IHC protocols and were excluded from this study, resulting in 124 appropriate cases for analysis.

Three pathologists (RSL, HG and CBN) all at once analyzed immunostained slides using a multi-head microscope and obtained a consensus score for HER2 expression according to Hofmann’s and Rüschoff’s recommendations for GC surgical specimens [11, 12]. The cases were categorized into four groups (0, 1+, 2+ and 3+), depending on the extension and intensity of the expression: 0, no reactivity, or membranous reactivity in < 10 % of cells; 1+, faint/barely perceptible membranous reactivity in > 10 % of cells, and cells are reactive only in part of their membrane; 2+, weak to moderate complete or basolateral membranous reactivity in > 10 % of cells; 3+, strong complete or basolateral membranous reactivity in > 10 % of cells [11]. Only a membranous pattern of expression was suitable for evaluation. The cases included into the 3+ category were considered positive for HER2 protein overexpression, whereas 0 and 1+ cases were regarded as negative.

Cases scored as 2+ were considered equivocal for HER2 protein expression and new 4 μm-thickness whole-tissue sections were submitted to silver brightfield *in situ* hybridization (SISH) using the same automated platform (BenchMark XT™) and the INFORM HER2 Dual ISH DNA Probe Cocktail assay (Ventana Medical Systems, Arizona, USA). The *HER2* and chromosome 17 (CEP17) copy numbers were counted in 20 nonoverlapping nuclei concurrently by two observers (RSL and CBN). *HER2* genomic profile was defined as non-amplified if the mean *HER2*/CEP17 ratio was <2.0 and amplified if the *HER2*/CEP17 ratio was ≥2.0. If the ratio fell between 1.8 and 2.2, an additional 20 nuclei were scored and the overall ratio calculated. IHC 2+ cases showing gene amplification by SISH and IHC 3+ cases were both considered positive for HER2 final status [11, 12].

The statistical analysis was performed using the SPSS version 19.0 software (SPSS Inc., Chicago, IL). The Pearson chi-square test was used to correlate IHC results and HER2 final status with clinicopathological parameters. All *P* values were two-sided, and *P* < 0.05 was considered statistically significant.

## Results

Clinicopathological characteristics and their association with HER2 final status and IHC scores are shown in Table 1. Patients’ age at diagnosis ranged from 28 to 92 years (median: 62.33 years), and there were 64 males (51.6 %) and 60 females (48.4 %) among 124 patients included in this study. Tumors were preferably identified at the distal stomach (80.6 %) in comparison with tumors arising in the gastric cardia or from the GEJ. Tumor size varied between 0.5 and 14.0 cm (average, 5.31 cm) and advanced tumors (pT2, pT3 and pT4 categories) accounted for 107 cases (86.3 %). According to histological grading, 13 cases (10.5 %) were well-differentiated, 45 cases (36.3 %) moderately differentiated, and 66 cases (53.2 %) poorly differentiated tumors. Laurén’s intestinal histological subtype was the most frequently diagnosed, accounting for 61 cases (49.2 %). Diffuse and mixed subtypes were observed in 21 (16.9 %) and 33 (26.6 %) cases, respectively. The nine remaining cases (7.3 %) were classified as indeterminate. Lymphovascular invasion was found in 94 cases (75.8 %), and 85 patients (68.5 %) had lymph node metastasis at diagnosis, with an average of 9.27 positive nodes per case (range, 1 to 63 positive nodes).

**Table 1:** Association between HER2 status and clinicopathological parameters

	Overall	HER2 positive	HER2 negative	*P* value	HER2 (3+)	HER2 (2+)	HER2 (0, 1+)	*P* value
	*n =* 124 (%)	13(10.5)	111 (89.5)		13(10.5)	10 (8.1)	101(81.4)	
**Age at diagnosis**				0.803				0.590
≥60 years	82 (66.1)	9	73		9	8	65	
<60 years	42 (33.9)	4	38		4	2	36	
**Gender**				0.865				0.459
Male	64 (51.6)	7	57		7	7	50	
Female	60 (48.4)	6	54		6	3	51	
**Tumor topography**				0.720				0.709
Distal stomach	100(80.6)	10	90		10	9	81	
Proximal stomach/GEJ	24 (19.4)	3	21		3	1	20	
**Histological subtype**				**0.048**				**0.009**
Intestinal	61 (49.2)	11	50		11	9	41	
Diffuse	21 (16.9)	0	21		0	0	21	
Mixed	33 (26.6)	2	31		2	1	30	
Unditermined	9 (7.3)	0	9		0	0	9	
**Histological grade**				**0.004**				**<0.001**
Well	13 (10.5)	4	9		4	1	8	
Moderately	45 (36.3)	7	38		7	8	30	
Poorly	66 (53.2)	2	64		2	1	63	
**Lymphovascular invasion**				**0.031**				0.060
Positive	94 (75.8)	13	81		13	6	75	
Negative	30 (24.2)	0	30		0	4	26	
**Blood-vessel invasion**				0.395				0.170
Positive	44 (35.5)	6	38		6	1	37	
Negative	80 (64.5)	7	73		7	9	64	
**pT status**				0.271				0.165
pT1	17 (13.7)	0	17		0	1	16	
pT2	32 (25.8)	3	29		3	2	27	
pT3	69 (55.6)	10	59		10	5	54	
pT4	6 (4.9)	0	6		0	2	4	
**pN status**				0.453				0.762
pN0	39 (31.5)	2	37		2	4	33	
pN1	46 (37.1)	7	39		7	4	35	
pN2	24 (19.3)	3	21		3	1	20	
pN3	15 (12.1)	1	14		1	1	13	
**pM status**				0.548				0.705
pM0	121(97.6)	13	108		13	10	98	
pM1	3 (2.4)	0	3		0	0	3	

Bold numbers represent *P* values with statistical significance

**Fig. 1:**
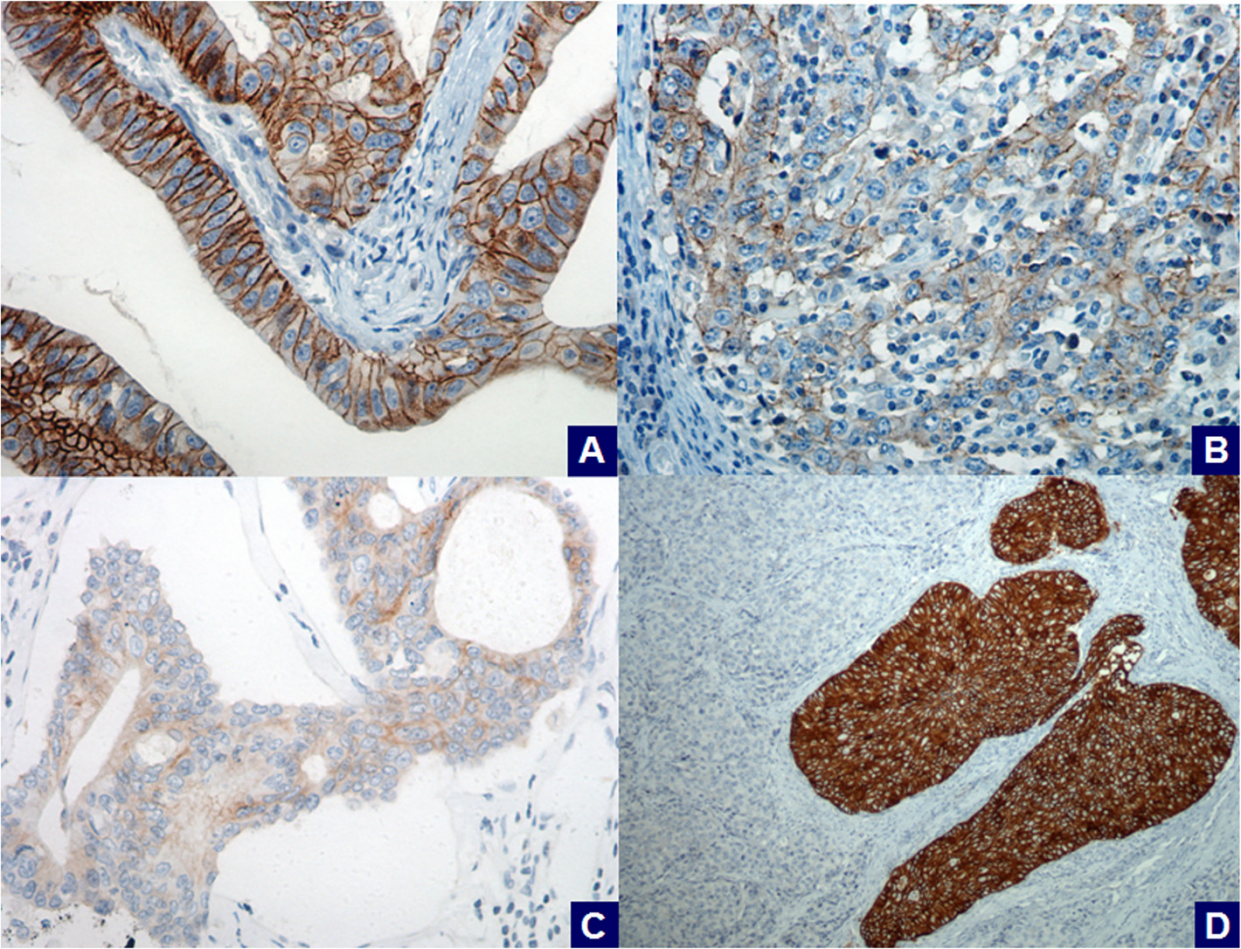
Immunohistochemical analysis of HER2 expression in gastric carcinomas using the 4B5 antibody in whole-tissue sections. (**a**) a HER2-positive case (3+); (**b**) a HER2-equivocal case (2+); (**c**) a HER2-negative case (1+); (**d**) intratumoral protein heterogeneity in a HER2-positive case (3+). (A-C: x400 field magnification; D: x200 field magnification)

Immunohistochemical analysis revealed 13 positive cases (3+; 10.5 %) for HER2 protein overexpression (Fig. 1a), 10 equivocal cases (2+; 8.1 %) (Fig. 1b) and 101 negative cases (81.4 %), 7 of which were scored 1+ (5.6 %) (Fig. 1c). Median age at diagnosis tended to increase according to HER2 status (negative, 61.76 years; equivocal, 66.3 years; and positive, 68.58 years), although this did not reach statistical significance. None of the patients in the IHC 3+ category were younger than 46 years of age and the oldest patient in the series (a 92 year old woman) was among them. There was no association between HER2 expression and patients’ gender.

There was a positive association between HER2 protein expression and well to moderatly differentiated tumors (*P* < 0.001), as well as with Laurén’s intestinal subtype (*P* = 0.009).

Tumors arising in the distal stomach accounted for 76.9 % of IHC 3+ cases, while 23.1 % where found in the gastric cardia or in the GEJ, but this variation was not statistically significant. No IHC 3+ tumor showed a diffuse or indeterminate morphology and only two (15.4 %) were considered poorly differentiated carcinomas. Every IHC 3+ tumor showed lymphovascular invasion and they were all diagnosed at an advanced stage of disease, mainly in the pT3 category (76.9 %). Eleven cases (84.6 %) were positive for lymph node metastasis, with an average of 7.5 positive nodes per case. However, no statistical significance was observed between HER2 protein overexpression and pT, pN or pM status.

Compared to IHC 3+ cases, HER2-equivocal tumors (2+) presented a slightly similar profile when morphological data was taken into account. No case showed diffuse or indeterminate histology, with a predominance of intestinal subtype (9 cases, 90.0 %) and moderately differentiated tumors (8 cases, 80.0 %). The majority of tumors in this setting also compromised the distal stomach and were diagnosed at an advanced stage of disease (9 cases, 90.0 %). However, there was a decrease in the proportion of cases showing lymphovascular invasion and lymph node metastasis (6 cases, 60.0 %) in comparison with numbers found among IHC 3+ cases (13 cases, 100.0 % and 11 cases, 84.6 %, respectively).

HER2-negative carcinomas (scores 0 and 1+) aggregated all tumors with a diffuse or indeterminate morphology as well as the majority of those with a poorly differentiated histological grade and early stage of the disease at diagnosis.

IHC 2+ cases were submitted to SISH but no gene amplification was detected. Therefore, cases with a final HER2-positive status were exclusively composed by IHC 3+ tumors, representing 10.5 % of all GC in this series. HER2 positivity was associated with intestinal subtype (*P* = 0.048), well to moderately differentiated tumors (*P* = 0.004) and presence of lymphovascular invasion (*P* = 0.031).

Regarding intratumoral HER2 protein heterogeneity, the extension of IHC 3+ areas ranged from 10 to 99 % of positive neoplastic cells (Fig. 1d). There was also a variation in the extension of HER2 immunoreactive areas in the equivocal category, ranging from 10 to 40 % of tumor cells. In both categories, intratumoral protein heterogeneity was mostly seen in mixed type carcinomas, in which HER2 expression highly correlated with intestinal-type areas.

**Fig. 2:**
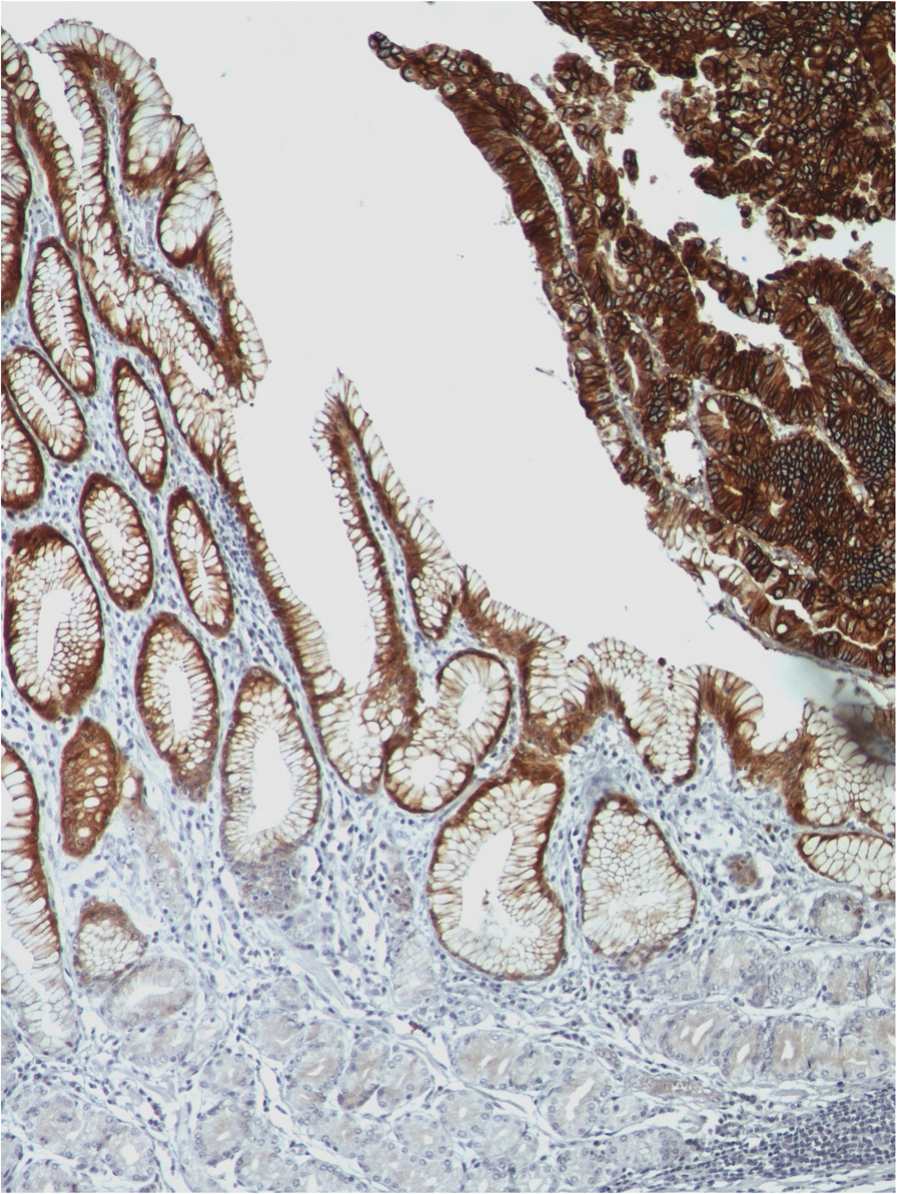
Possible pitfalls in the immunohistochemical analysis of HER2 protein expression using the 4B5 antibody. Cytoplasmic non specific staining in normal foveolar gastric mucosa (left) and in a *focus* of gastric high-grade dysplasia (right) (x200 field magnification)

Apart from their HER2 score, many cases displayed a cytoplasmic immunoreactivity in normal gastric mucosa cells (87 cases, 70.2 %), metaplastic epithelium (30 cases, 24.2 %) and *foci* of dysplasia (24 cases, 19.4 %) (Fig. 2). This pattern of expression was seen in both metaplastic and dysplastic epithelium, despite the nature of the metaplasia or the degree of the dysplasia. Nuclear expression was frequently seen among diffuse type carcinomas, but all these immunostained areas were not taken into account for the scoring of the HER2 immunohistochemical profile.

## Discussion

In our study, we found a final HER2-positive status of 10.5 %. The overall HER2 positivity rate found in the ToGA study was 810 of 3,665 patients (22.1 %) [6]. These numbers represent one of the largest sets of HER2 testing data in GC samples, obtained from patients from 24 different countries in Asia, Europe and Latin America. Although 22.1 % accounts for more than double the amount of positive tumors we found in our series, the ToGA trial HER2 positivity screening criteria included all cases with protein expression by IHC (3+ and 2+ cases) or gene amplification by ISH alone. More detailed data concerning HER2 testing in the ToGA study has been recently published revealing that precisely 610 cases out of 3,665 tumor samples (16.6 %) were in fact considered HER2 positive, by either scoring IHC 3+ or IHC 2+ with gene amplification by fluorescence *in situ* hybridization (FISH) [30]. A *post hoc* exploratory analysis concluded that patients in those categories were the ones who utterly benefited the most from treatment with TZB [6]. Thus, the exact percentage of patients eligible for treatment with TZB according to HER2 screening performed on the ToGA trial was 16.6 % and, when IHC results are considered alone, only 398 cases (10.9 %) were actually HER2 positive (3+) [30]. In our study, since no IHC 2+ cases showed gene amplification by SISH, we found a IHC 3+ rate and a final HER2 positive status of 10.5 %.

GC in Brazilian patients tends to arise in the antrum, associated with *Helicobacter pylori* chronic infection, therefore, our series is composed mainly of carcinomas of the distal stomach (80.6 %). Since HER2 expression has been greatly associated with GC of the cardia and GEJ [17, 30–33], we expected to find an even lower positivity rate among our cases. ToGA trial results showed a difference in HER2 positivity rates between Europe/Asia and Central/South America (23.8 and 16.1 % respectively, including all FISH-positive and IHC 3+ cases). The authors associated the contrasting results as due to the lower number of patients screened in Latin America (484 out of 3,665 patients) instead of a possible variation in tumor biology [30]. Unfortunately, we could not find information specifically regarding HER2 expression in GC of Brazilian patients enrolled in the ToGA study. If cross-data between tumor topography and patients’ countries of origin could be retrieved, it might also help explain divergent HER2-positive GC rates in Latin America.

Similar to our results, in a recent large European series [34], as well as in a Japanese cohort of 1,461 patients [35], there was no difference in HER2 positivity between proximal and distal gastric tumors. However, two large Chinese series [17, 33] recently showed significantly higher HER2 expression in proximal GC, in agreement with ToGA trial findings [30] and with results from a series of 2,798 South Korean patients [32]. The authors have implied that intestinal-type cancers are usually more frequent in the GEJ and, as different etiologies may play a role in carcinogenesis of tumors from these two locations, this could partially justify distinct HER2 expression rates according to tumor topography [30]. In addition to the genetic ancestry according to ethnicity, multiple gastric carcinogenesis pathways may be greatly influenced by dietary habits and chronic *Helicobacter pylori* infection [3]. Although this probably also has some influence on HER2 positivity rates found in different populations with GC, the relation between these factors and HER2 expression remains unknown, with few papers published [36, 37].

The number of IHC 3+ cases found in our study could be explained in part by the fact that whole-tissue sections were exclusively used for HER2 evaluation, instead of endoscopic biopsies. A recent study paired primary gastrectomies and their endoscopic biopsies to compare HER2 expression scores and found a concordance rate of only 57 %, mostly due to intratumoral HER2 heterogeneity [38]. HER2 genetic heterogeneity by ISH has been previously demonstrated on breast cancer, but it seems to be a more complex issue on GC specimens [39]. Protein expression heterogeneity by IHC, usually referred as variability in immunohistochemical intensity and extension of HER2-positive areas, is also a problem which could greatly impact on sample selection for HER2 testing in GC. In fact, HER2 expression heterogeneity has been demonstrated not only by our study but also by other authors [39, 40], including a study comprising 2,727 gastrectomies [32]. As a result, whole-tissue sections obtained from resected primary gastric tumors may offer a larger representation of different sub clonal cancer cell population which could help avoid false-negative results due to HER2 heterogeneity. Since patients with locally advanced or metastatic GC might not undergo primary gastrectomy, whole-tissue sections are not always available and other GC samples ought to be used instead, raising the question as to which are the best suited for HER2 testing: endoscopic biopsies *versus* metastatic lesions. For endoscopic biopsies, the recommendation is a viable number of representative tumor fragments (ideally 6–8) and, if possible, the pathologist should also perform HER2 testing on another specimen when a negative result is found on a tumor endoscopic biopsy [40].

In addition, GC samples from the ToGA trial were tested for HER2 expression with the HercepTest kit, which uses a rabbit polyclonal anti-HER2 antibody [6], whereas a rabbit monoclonal antibody (4B5) was employed in our study. Other studies have reported lower sensitivity rates for HercepTest in comparison with 4B5 clone for the detection of HER2 protein expression [15, 41, 42]. This could also account for the similar IHC 3+ percentage of cases found in our series and on the ToGA trial, despite the predominance of tumors from the distal stomach among our patients.

Data on HER2 expression in Brazilian patients with GC are very limited and there is a wide variation on published results. The first two studies ever conducted in Brazil found a HER2 overexpression rate of 12 and 5.4 %, including IHC 3+ and 2+ cases added together [23, 24]. However, the grading score for breast cancer was used for HER2 status assessment, in which the entire cell membrane should be stained, and not only lateral or basolateral walls, which suggests underestimation of HER2 positivity. In more recent research using specific HER2 scoring criteria for GC, IHC 3+ cases ranged from 0.8 % to 11.6 % [19–22, 25, 26]. Studies showing the lowest rates (0.8 %, 3 % and 6 %) used tumor tissue microarrays (TMAs) obtained from surgical specimens and other anti-HER2 antibodies rather than 4B5 [19, 20, 22]. These results are difficult to be compared with ours due to methodological differences, especially when TMAs and tumoral protein expression heterogeneity are confronted. Thus, TMAs could represent samples more similar to endoscopic biopsies rather than to larger whole-tissue sections from primary gastrectomies, which were used in our study.

Our results corroborate current literature findings on Brazilian patients with GC tested for HER2 expression using a highly sensitive anti-HER2 antibody in whole-tissue tumor sections. As a matter of fact, a recent analysis from a Brazilian cancer center compared the performance of HercepTest, SP3 and 4B5 antibodies between whole-tissue sections and TMAs in a series of 199 gastrectomies [21]. The authors found the highest HER2 positivity rate using the same parameters of our study: 4B5 antibody in whole-tissue tumor sections (11.6 %), a value most similar to our finding (10.5 %). The lowest HER2 positivity rate was identified among TMAs and HercepTest kit (3 %) [21].

Despite the higher sensitivity found with the 4B5 antibody, some authors have also reported extensive cytoplasmic background staining of the gastric foveolar layer and *foci* of intestinal metaplasia/dysplasia, as seen in our study, which suggests an intrinsic clonal characteristic rather than a methodological problem [15, 21, 41, 42]. In fact, HER2 staining of dysplastic epithelium has been correlated to gene amplification by ISH [43]. Although there were no difficulties in distinguishing HER2-positive neoplastic cells from those areas, we stress that the staining of the latter must not be considered when scoring HER2 expression.

Until the writing of the present manuscript the authors were able to retrieve only three other studies regarding ISH for gene amplification detection in GC samples from Brazilian patients [19, 21, 25]. Begnami et al. found an overall amplification rate of 8 % using FISH, but the percentage of IHC 2+ amplified cases was not mentioned [19]. Similar to our findings, another group did not find any amplified cases among IHC 2+ tumors, although FISH was the employed method [25]. Besides our study, only another one applied silver brightfield dual ISH in IHC 2+ cases [21]. Results showed a wide range of amplification rates (from zero to 100 %), according to the primary antibody used for IHC and tissue samples (whole-tissue sections *versus* TMAs). 4B5 antibody on 198 whole-tissue sections revealed 20 cases with a equivocal HER2 score (10.1 %) of which only 6 cases (30 %) showed gene amplification by SISH [21]. The percentage of cases with a final HER2 positive status was 14.6 %, a slightly higher rate than the one we uncovered, which could be partly due to the lower frequency of distal GC (43.2 %) in their series. They have concluded that the most accurate IHC method for evaluating HER2 expression in GC was to use the 4B5 antibody on whole-tissue sections [21], which was exactly the method employed in our study.

Moreover, we found an association between HER2 positive tumors and some pathological parameters, such as Laurén’s intestinal subtype (*P* = 0.048), well to moderately differentiated tumors (*P* = 0.004) and presence of lymphovascular invasion (*P* = 0.031). The intestinal subtype has been proven to be the pathological feature most invariably associated with HER2 positivity in multiple studies [17, 32, 34, 35, 44, 45], including the ToGA trial [30] and some with Brazilian patients [19, 22, 23, 26]. Since HER2 positivity is expected to be found on intestinal-type tumors, we strongly encourage that, when dealing with a mixed-type tumor, areas showing an intestinal morphology should be selected for HER2 scoring.

Association of HER2 positivity with histological grade and presence of lymphovascular invasion has also been reported by some authors [19, 25, 31, 46] but not by others [20, 47]. A recent meta-analysis comprising 15 studies and 5,290 patients concluded that not only Laurén’s classification system but also tumor differentiation and lymphovascular invasion were associated with HER2 positivy [48]. Table 2 summarizes results from various studies concerning HER2 positivity rates and the association between HER2 expression with clinicopathological features in patients with GC from different parts of the world, in comparison to our findings.

**Table 2:** HER2 expression in gastric cancer: analysing studies with patients from different parts of the world

Study	Year	Patients’ place of origin	Number of patients	HER2 positive rate (%)	Association with clinicopathological features
Bang YJ [6]	2010	ToGA trial (Multicenter)	3,665	16.6	Topography (GEJ)
					Intestinal-type
Begnami MD [19]	2011	Brazil (Single center)	221	8.0	Intestinal-type
					Low-grade
Cruz-Reyes C [45]	2013	Mexico (Single center)	269	3.7	Intestinal-type
Cho J [32]	2013	South Korea (Single center)	2,798	7.3	Older age
					Male gender
					Intestinal-type
					Upper-third stomach
					Higher lymph node stage
					Advanced staging
Shan L [17]	2013	China (Single center)	1,463	9.8	Topography (GEJ)
					Intestinal-type
					Low-grade
Matsusaka S [35]	2015	Japan (Multicenter)	1,461	15.6	Intestinal-type
					Hepatic metastasis
					Absence of peritoneal metastasis
Cappellesso R [34]	2015	Europe (Multicenter)	1,040	11.0	Intestinal-type
					Low-grade
Laboissiere RS	2015	Brazil (Single Center)	124	10.5	Intestinal-type
					Low-grade
					Lymphovascular invasion

*GEJ* gastroesophageal junction

## Conclusions

Our study demonstrates a HER2-positive GC frequency of 10.5 % among Brazilian patients, analogous to rates observed in different populations from studies applying similar methodology. Also, our findings showed an association of HER2 positive status with Laurén’s intestinal subtype, well to moderately differentiated tumors, and presence of lymphovascular invasion, as it has been reported by other authors. Finally, there was no association found with tumor topography mainly due to the predominance of carcinomas of the gastric antrum in our series, as expected among Brazilian patients with GC.

## Abbreviations

CEP17: chromosome 17
FISH: fluorescence *in situ* hybridization
GC: gastric cancer
GEJ: gastroesophageal junction
IHC: immunohistochemistry
ISH: *in situ* hybridization
SISH: silver *in situ* hybridization
TMA: tissue microarray
TZB: trastuzumab
